# Circular RNA cir-ITCH Is a Potential Therapeutic Target for the Treatment of Castration-Resistant Prostate Cancer

**DOI:** 10.1155/2020/7586521

**Published:** 2020-08-20

**Authors:** Shoubin Li, Chunhong Yu, Yunxia Zhang, Junjiang Liu, Yi Jia, Fuzhen Sun, Panying Zhang, Jingpo Li, Liuxiong Guo, Helong Xiao, Fei Gao, Xinna Deng, Ziqi Cai, Jianhui Cai

**Affiliations:** ^1^Department of Surgery, Hebei Medical University, Shijiazhuang, Hebei 050017, China; ^2^Department of Urology, Health Examination Center, Obstetrics and Gynecology, and Oncology, Hebei General Hospital, Shijiazhuang, Hebei 050051, China; ^3^Hebei Engineering Technology Research Center for Cell Therapy, Hebei HOFOY Biotech Corporation Ltd., Shijiazhuang, Hebei 050000, China

## Abstract

cir-ITCH, a well-known tumor-suppressive circular RNA, plays a critical role in different cancers. However, its expression and functional role in prostate cancer (PCa) are unclear. Herein, we explored the potential mechanism and tumor-inhibiting role of cir-ITCH in PCa. Using reverse transcriptase polymerase chain reaction assay, we analyzed the expression of cir-ITCH in PCa and paired adjacent nontumor tissue samples resected during surgical operation, as well as in two cell lines of human PCa (LNCaP and PC-3) and the immortalized normal prostate epithelial cell line (RWPE-1). Cell viability and migration of PCa cell lines were evaluated using CCK-8 and wound-healing assays. Expression of key proteins of the Wnt/*β*-catenin and PI3K/AKT/mTOR pathways was detected using western blotting. We found that cir-ITCH expression was typically downregulated in the tissues and cell lines of PCa compared to that in the peritumoral tissue and in RWPE-1 cells, respectively. The results showed that cir-ITCH overexpression significantly inhibits the proliferation, migration, and invasion of human PCa cells and that reciprocal inhibition of expression occurred between cir-ITCH and miR-17. Proteins in the Wnt/*β*-catenin and PI3K/AKT/mTOR pathways were downregulated by overexpression of cir-ITCH both in androgen receptor-positive LNCaP cells and androgen receptor-negative PC-3 cells. Taken together, these data demonstrated that cir-ITCH plays a tumor-suppressive role in human PCa cells, partly through the Wnt/*β*-catenin and PI3K/AKT/mTOR pathways. Thus, cir-ITCH may serve as a novel therapeutic target for the treatment of PCa, especially castration-resistant prostate cancer.

## 1. Introduction

Since the androgen receptor (AR) signaling plays a critical role in the development and progression of prostate cancer (PCa), androgen deprivation therapy (ADT) or AR antagonists remain the standard treatment for PCa patients. However, almost all patients eventually progress to castration-resistant prostate cancer (CRPC), which is resistant to therapy [1]. The main factors accountable for the growth of CRPC are relevant to the AR pathway [2–4]. Although nearly half of the men with CRPC respond to novel hormonal agents such as abiraterone or enzalutamide, AR splice variants may still cause resistance [5]. Therefore, new targets independent of AR are necessary for the treatment of CRPC.

Circular RNA (circRNA) is a novel class of endogenous noncoding RNAs formed by a covalently closed loop [6]. Current evidence revealed that circRNAs may enhance or suppress cancer progression by suppressing miRNA species associated with the development of tumors [7].

cir-ITCH is a well-known circRNA that sponges different miRNAs and regulates the Wnt/*β*-catenin pathway. In addition, it has an inhibitory role in the malignancies of the colorectum [8], lung [9], esophagus [10], and bladder [11]. However, its expression and functional role in prostate cancer, especially in CRPC, are not well demonstrated. We thus clarified its role in two different PCa cell lines (LNCaP and PC-3) representing hormone-sensitive PCa and CRPC, respectively.

## 2. Materials and Methods

### 2.1. Clinical Samples

Ten pairs of primary prostate cancer tissues and matched adjacent nontumor tissue samples were obtained from the Department of Urology, Hebei General Hospital, between January and December 2018, and were snap-frozen in liquid nitrogen and stored at -80°C until RNA extraction. All patients involved in the study provided their written informed consent. The study protocol was approved by the Ethics Committee of Hebei General Hospital.

### 2.2. Cell Culture

An immortalized normal prostate epithelial cell line (RWPE-1) and two PCa cell lines (LNCaP, PC-3) were purchased from the Chinese Academy of Sciences. The two PCa cell lines were cultured in RPMI-1640 (Gibco, Grand Island, NY, USA) containing 10% fetal bovine serum while RWPE-1 cells were cultured in Prostate Epithelial Cell Medium-PRF (ScienCell, Carlsbad, CA, USA). All cells were incubated at 37°C with an atmosphere with 5% CO2.

### 2.3. Circular RNA Plasmid Construction and Transient Transfections

**Figure 1:**
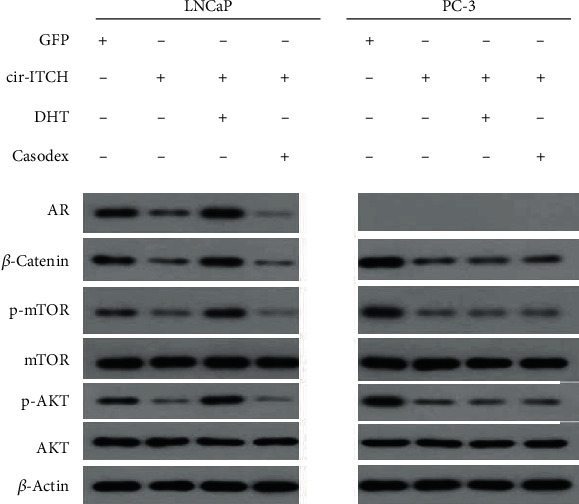
AR expression is not expressed in PC-3 cells. cir-ITCH downregulated the protein expression level of *β*-catenin, p-AKT, and p-mTOR in LNCaP cells and PC-3 cells as seen with western blot. The expression of representative proteins in PC-3 cells was not affected by exogenous androgen or androgen receptor antagonist.

To overexpress human cir-ITCH (hsa_circ_0001141), cDNA was synthesized by GenScript Biotech (Nanjing, China) and cloned into pLC5-ciR (Geneseed Biotech, Guangzhou, China). Lentivirus was constructed by Wanleibio (Shenyang, China). For cell proliferation, qRT-PCR, migration, and invasion assays and experiments to verify the interaction between cir-ITCH and miR-17-5p, LNCaP, and PC-3 cells were treated with different transient transfections agents (GFP, cir-ITCH, miR-17, and cir-ITCH + miR-17). For immunoblot, LNCaP and PC-3 cells were divided into four groups according to different treatments (Figure 1). The dosage of dihydrotestosterone (DHT) was 10 nM and that of Bicalutamide (Casodex) was 10 *μ*M. Transfection was performed as previously described according to the manufacturer's instructions [12].

### 2.4. RNA Extraction and Quantitative Real-Time Polymerase Chain Reaction (qRT-PCR)

Total RNA was isolated from tissues and cells using TriPure reagent (BioTeke, Beijing, China), according to the manufacturer's protocol. The concentration of RNA in each sample was determined by ultraviolet spectrophotometer NanoDrop 2000 (Thermo Fisher Scientific, Waltham, MA, USA). cDNA was synthesized using Super M-MLV reverse transcriptase (BioTeke, Beijing, China), and qRT-PCR for circRNA was performed on Exicycler 96 (Bioneer, Seoul, Korea) using primers (Sango Biotech, China) listed in Table 1. U6 and *β*-actin were used as internal controls for miRNA and mRNA detection, respectively.

Each sample was replicated three times, and data was analyzed by comparing CT values [13].

### 2.5. Cell Proliferation Assay

Transfected cells were seeded into 96-well plates for cell proliferation experiments (4000 cells per well). Cell viability was measured using the cell counting kit 8 (CCK-8) system (Wanleibio, Shenyang, China), 0, 24, 48, and 72 hours after seeding. Each group consisted of six replicates. Three independent experiments were performed.

### 2.6. Migration and Invasion Assays

The wound-healing assay was conducted 24 hours after transfection to evaluate cell migration. A linear wound was created with a pipette tip across the confluent cell layer 1 h after treatment with 1 *μ*g/mL mitomycin C. After two washing steps with serum-free medium, the cells were cultured at 37°C in an incubator in the presence of 5% CO2 for 24 h. Wound sizes were observed and measured before incubation and 24 h later.

In the invasion assay, the method described in a previous study [11] was used. Approximately 2 × 10^4^ transfected cells were seeded into the upper chambers of each Transwell, which was coated with Matrigel (Corning, USA). Medium containing 30% FBS was added to the lower chamber. The cells were incubated at 37°C with 5% CO2 for 24 h. After incubation, the cells in the top chamber were wiped off with a cotton swab, and the cells on the lower surface were fixed with methanol, stained with 0.1% crystal violet, and photographed at 100x magnification under a microscope (Olympus, Japan). The number of cells in each sample was counted in five visual fields, and the average number was recorded.

### 2.7. Western Blotting

Protein extracts were obtained from cell lysates and quantified using the bicinchoninic acid (BCA) analysis method (Wanleibio, Shenyang, China). Proteins were separated by 10% SDS-PAGE and transferred to polyvinylidene fluoride (PVDF) membranes. (Merck Millipore, Billerica, MA, USA). After incubation with high-affinity antibodies (anti-p21 (1 : 500), anti-PTEN (1 : 500), anti- AR (1 : 500), anti-mTOR (1 : 500), anti-p-mTOR (1 : 500), anti-AKT (1 : 500), anti-p-AKT (1 : 500), and anti-*β*-actin antibody (1 : 1000) (Wanleibio, Shenyang, China), the membranes were incubated with a secondary antibody (1 : 5000, Wanleibio, Shenyang, China). After incubation, ECL luminescent solution (Wanleibio, Shenyang, China) was evenly added to the PVDF membranes, these membranes were loaded onto the cassette and exposed in a darkroom. After washing, signals were detected and analyzed using the Gel-Pro-Analyzer software.

### 2.8. Statistical Analysis

The SPSS 19.0 software (SPSS Inc., Chicago, IL, USA) was used for statistical analysis. The data were expressed as mean ± SD. Data differences between two groups were analyzed using the Student's *t*-test. A significance level was defined as *P* < 0.05.

## 3. Results

### 3.1. cir-ITCH Is Downregulated in PCa

**Figure 2:**
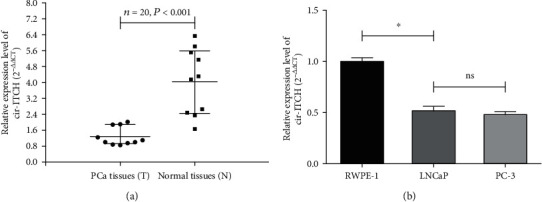
cir-ITCH expression was significantly decreased in PCa tissues and PCa cell lines. In (a), relative expression levels of cir-ITCH in PCa tissues and adjacent normal tissues were detected by qRT-PCR (*P* < 0.001, Student's *t*-test). In (b), relative expression levels of cir-ITCH in prostate cancer cell lines and RWPE-1 cell line were detected by qRT-PCR (∗*P* < 0.05; ns: no significance, Student's *t*-test).

Firstly, we examined the expression of cir-ITCH in 10 pairs of PCa tissues and matched adjacent normal tissues using qRT-PCR. Results indicated that cir-ITCH expression was significantly decreased in PCa tissues, compared to that in the matched adjacent normal tissues (Figure 2(a)). Additionally, cir-ITCH expression was lower in LNCaP and PC-3 cells, compared to the RWPE-1 cell line (Figure 2(b)). Of note, there was no significant difference in the expression of cir-ITCH between the two prostate cancer cell lines.

### 3.2. cir-ITCH and miR-17 Negatively Interacts in PCa

**Figure 3:**
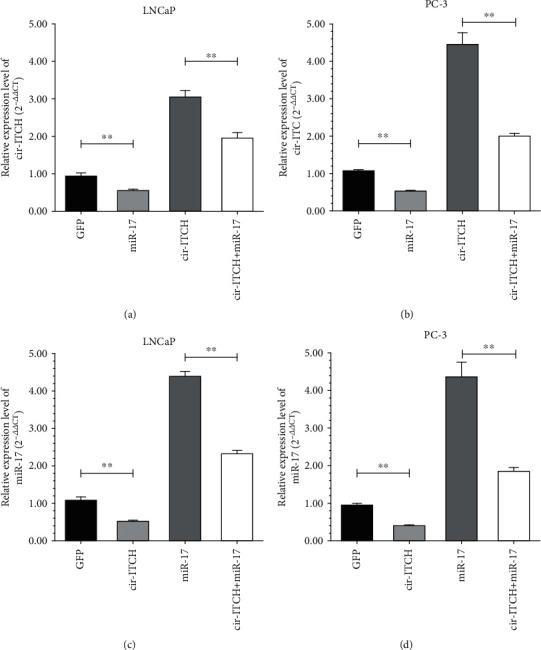
cir-ITCH and miR-17 inhibited each other. The expression of cir-ITCH in LNCaP cells (a) and PC-3 cells (b) treated with different transfection agents (GFP, miR-17, cir-ITCH, and cir-ITCH + miR-17) (∗∗*P* < 0.01, Student's *t*-test). The expression of miR-17 in LNCaP cells (c) and PC-3 cells (d) treated with different transfection agents (GFP, cir-ITCH, miR-17, and cir-ITCH + miR-17) (∗∗*P* < 0.01, Student's *t*-test).

To verify the interaction between cir-ITCH and miR-17, we examined the expression of cir-ITCH or miR-17 in LNCaP and PC-3 cells treated with different agents of transfection using qRT-PCR. As shown in Figure 3, we observed a reciprocal inhibition between cir-ITCH and miR-17. Compared with GFP transfection, miR-17 significantly decreased the expression of cir-ITCH in LNCaP and PC-3 cells. Furthermore, cotransfection with miR-17 and cir-ITCH significantly attenuated the enhancing effect caused by transfection with cir-ITCH alone (Figures 3(a) and 3(b)). In turn, overexpression of cir-ITCH inhibited the expression of miR-17 and counteracted its effect in both LNCaP and PC-3 cells (Figures 3(c) and 3(d)).

### 3.3. cir-ITCH Inhibits the Progression of PCa Cells In Vitro

**Figure 4:**
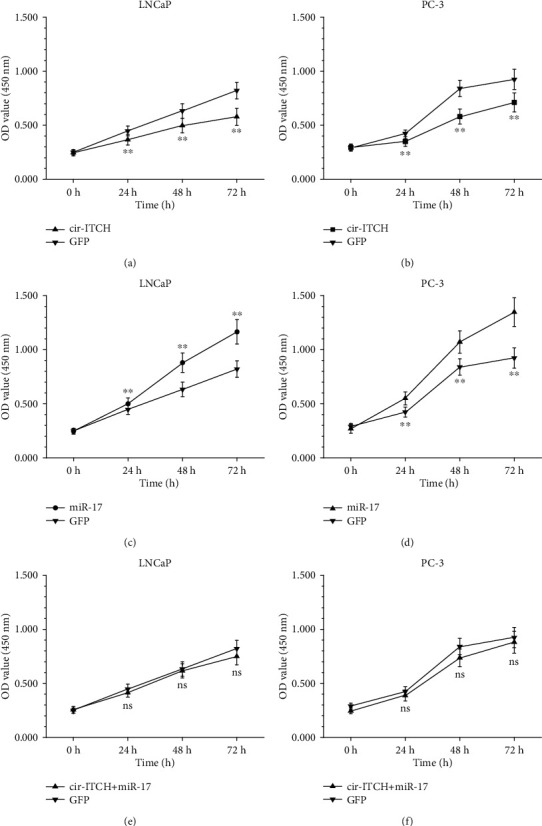
The effects of cir-ITCH or miR-17 on the viability of PCa cell lines. Overexpression of cir-ITCH significantly inhibited the viabilities of LNCaP (a) and PC-3 (b) cells compared with GFP-transfected cells (∗∗*P* < 0.01, Student's *t*-test). miR-17 significantly promoted the viabilities of LNCaP (c) and PC-3 (d) cells (∗∗*P* < 0.01, Student's *t*-test). Viabilities of PCa cells cotransfected with cir-ITCH and miR-17 were comparable to viabilities of GFP-transfected cells (e, f) (ns: no significance, Student's *t*-test).

**Figure 5:**
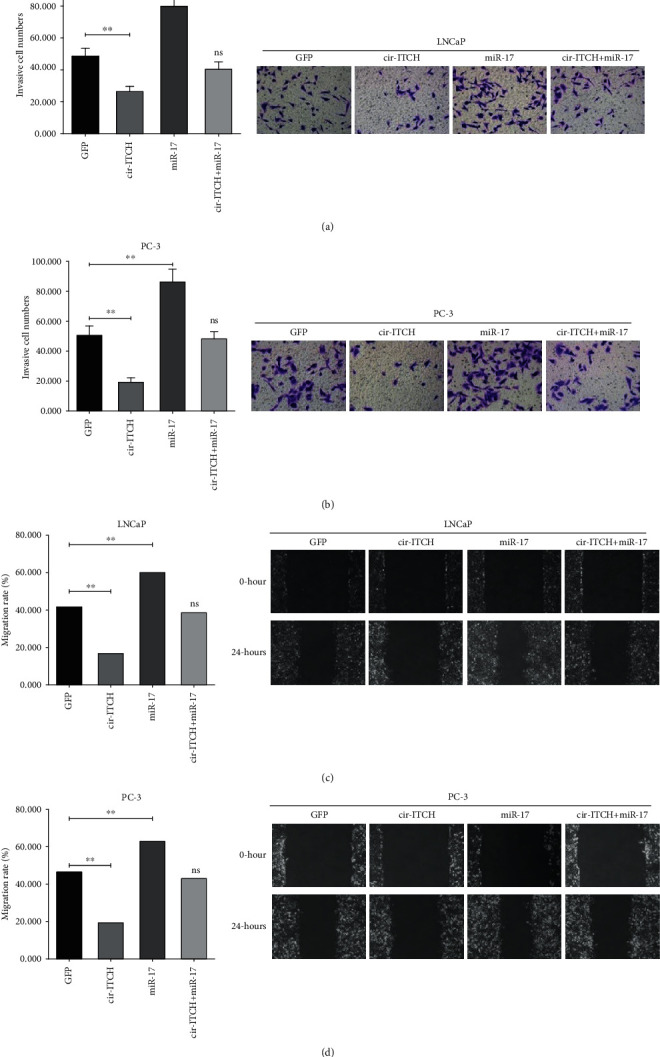
Transwell invasion assay results expressed as the number of invaded cells per field. cir-ITCH inhibited the invasion abilities of LNCaP cells (a) and PC-3 cells (b) compared with GFP controls, while miR-17 promoted the invasion abilities and counteracted the effect of cir-ITCH. No significant difference was observed in the context of cotransfection (cir-ITCH + miR-17) and the GFP control (magnification, 100x, ∗∗*P* < 0.01; ns: no significance, Student's *t*-test). Wound-healing assay results were expressed as migration rates. The results showed that overexpression of cir-ITCH resulted in the slower closure of scratch wounds in both LNCaP cells (c) and PC-3 cells (d), while transfection of miR-17 resulted in faster closure. Cotransfection with miR-17 and cir-ITCH resulted in an effect comparable to the GFP control condition (∗∗*P* < 0.01; ns: no significance, Student's *t*-test).

Functionally, CCK-8 assays revealed that the viability of LNCaP and PC-3 cells decreased in the cir-ITCH overexpression group compared with that in the GFP group (Figures 4(a) and 4(b)). Transfection of miR-17 significantly promoted the viability of both cell lines (Figures 4(c) and 4(d)). The tumor-suppressive effect of cir-ITCH was blocked by miR-17 cotransfection (Figures 4(e) and 4(f)). Meanwhile, Transwell invasion assays indicated that the invasion abilities of LNCaP and PC-3 cell lines were suppressed by the overexpression of cir-ITCH, which was in accordance with the wound-healing assay results (Figure 5). miR-17 mimics could partly attenuate the inhibition of migration and invasion that was mediated by cir-ITCH overexpression in PCa cells.

### 3.4. cir-ITCH Is Involved in the Regulation of the Wnt/*β*-Catenin and PI3K/AKT/mTOR Signaling Pathways

We investigated key proteins of the pathways in which cir-ITCH may probably participate. Levels of *β*-catenin, AKT, p-AKT, mTOR, and p-mTOR in cells treated with different agents were analyzed using western blot. As shown in Figure 1, there was an obvious decrease in *β*-catenin, p-AKT, and p-mTOR in cir-ITCH-overexpressed prostate cancer cells, and in both androgen receptor-positive LNCaP cells and androgen receptor-negative PC-3 cells. However, there was no obvious change in AKT and mTOR expression in the different groups. Additionally, in LNCaP cells, DHT treatment increased the expression of *β*-catenin, p-AKT, and p-mTOR, while Casodex treatment inhibited the expression of these proteins. On the other hand, in PC3 cells, DHT and Casodex treatments did not impact the expression of these proteins.

## 4. Discussion

Most cases of prostate cancer will progress into CRPC after a period of androgen deprivation treatment, and the average survival for patients with CRPC is 2–3 years [14]. The mechanism of CRPC has not yet been fully demonstrated.

The Wnt/*β*-catenin and PI3K/AKT/mTOR pathways were reported to play critical roles in the growth of CRPC. *β*-Catenin can directly bind to AR to enhance its transcriptional activity in LNCaP cells [15]. Furthermore, nuclear *β*-catenin can augment the activity of AR in the absence of androgen [16]. *β*-Catenin is a coactivator of AR, and activation of Wnt/*β*-catenin signaling was thought to account for CRPC growth [17] and progress of prostate cancer [18]. Wnt/*β*-catenin inhibitor can increase sensitivity of androgen-independent prostate cancer cells to the second-generation androgen receptor antagonist, enzalutamide, suggesting the therapeutic potential of this approach [19, 20]. The phosphatidylinositol 3-kinase (PI3K)/AKT pathway is another important signaling pathway in prostate carcinogenesis [21]. AKT and mTOR are important downstream targets of PI3K, activated by phosphorylation. Studies have shown that activation of the PI3K/AKT signaling pathway can promote prostate cancer cell invasion [22] and the PI3K/AKT/mTOR pathway is associated with advanced prostate cancer and bone metastasis [23]. Notably, there is an interaction between the PI3K/AKT/mTOR and AR pathways. Long-term androgen deprivation treatment can lead to resistance of tumor cells to apoptosis because of the activation of the PI3K/AKT pathway [24]. On the other hand, inhibition of the PI3K/AKT pathway leads to the activation of the AR pathway. Thus, simultaneous inhibition of these two pathways has achieved significant antitumor effects [25].

Although the Wnt/*β*-catenin and PI3K/AKT/mTOR pathways are essential for the growth of CRPC, their roles still mainly rely on interaction with the androgen receptor axis. During the progression of CRPC, a subtype of PCa undergoes divergent clonal evolution and becomes truly androgen receptor-independent [26]. Therefore, novel-targeted treatments are needed.

Circular RNAs (circRNAs) were presented as a new class of pervasive RNA with regulatory potential; they can bind to microRNAs (miRs) as a competing endogenous RNA (ceRNA), thereby repressing miR function. Hundreds of circRNAs expressed in normal tissues and prostate cancer have been identified [27]. The higher expression of circ-MTO1 in tumor tissue compared to normal tissues suggests a better prognosis. Overexpression of circ-MTO1 in prostate cancer cells can inhibit cell viability and the expression of miR-17 [28]. circRNA circfoxo3 reportedly promotes the development of prostate cancer by sponging miR-29a-3p [29], while circSMARC5 is upregulated in prostate cancer and its expression is androgen-responsive [30].

cir-ITCH is a well-known tumor-suppressing circRNA generated from several exons of itchy E3 ubiquitin protein ligase (ITCH), which is downregulated in colorectal cancer [8], lung cancer [9], esophageal squamous cell carcinoma [10], and bladder cancer [11], and serves as a sponge for certain miRNAs, including miR-17 and miR-214 [11, 31]. However, the role of cir-ITCH in prostate cancer, especially in CRPC, has not been well described. In the present study, we found that cir-ITCH expression was significantly downregulated in both PCa tissues as well as PCa cell lines. Overexpression of cir-ITCH inhibited the growth, migration, and invasion of PCa cells, proving its tumor inhibitory function in PCa. Both tumor-promoting as well as tumor-suppressing roles in PCa have been reported for miR-17 [28, 32]. Evidence has shown that high or low miR-17 expression in the blood is related to the progression of PCa [33]. The results of the present study showed that miR-17 exerted a tumor-promoting effect in PCa that could be offset by cir-ITCH. Therefore, we can conclude that cir-ITCH possibly inhibits prostate cancer by sponging miR-17, which is consistent with the literature [34].

cir-ITCH plays a tumor-suppressing role through inhibition of the Wnt/*β*-catenin pathway in several neoplasms [8–11]. We found decreased expression of p-AKT and p-mTOR in cir-ITCH-overexpressing PCa cells, which indicated that cir-ITCH may also inhibit the PI3K/AKT/mTOR pathway. More importantly, cir-ITCH inhibited representative proteins of the Wnt/*β*-catenin and PI3K/AKT/mTOR pathways in PC3 cells, which do not express AR and represent androgen receptor-negative PCa. Thus, we expect future research efforts to explore cir-ITCH as a new potential therapeutic target in PCa.

## 5. Conclusions

Our study investigated the role and mechanism of cir-ITCH in PCa and provided evidence indicating that cir-ITCH functions as tumor suppressor in prostate cancer cells via the Wnt/*β*-catenin and PI3K/AKT/mTOR pathways in an AR-independent manner. Our findings may provide a potential therapeutic target for the management of PCa, especially CRPC.

## Figures and Tables

**Table 1:** The sequences of primers used in this study.

Gene	Forward (5′-3′)	Reverse (3′-5′)
hsa_circ_0001141	CAGCGTAGTCAGCTTCAA	GTTGGCTCTTTGTCACCT
*β*-Actin	CGGGAAATCGTGCGTGAC	GTCAGGCAGCTCGTAGCTCTT
hsa-miR-17-5p	CCAGCCAAAGTGCTTACAGTGC	GTGCAGGGTCCGAGGTATTC
U6	GCTTCGGCAGCACATATACT	GTGCAGGGTCCGAGGTATTC

## Data Availability

The data used to support the findings of this study are available from the corresponding author upon request.
